# The combined uro-radiological “Rendezvous” procedure, and an unusual CT and cystoscopic finding

**DOI:** 10.1259/bjrcr.20180026

**Published:** 2018-10-05

**Authors:** Robert Hodnett, Catherine Gutteridge, Ivo Dukić

**Affiliations:** 1 Peninsula Radiology Academy, Plymouth, UK; 2 Department of Radiology, Plymouth Hospitals NHS Trust, Plymouth, UK; 3 Department of Urology, Plymouth Hospitals NHS Trust, Plymouth, UK

## Abstract

The ureteric “rendezvous” procedure is an interventional procedure involving both uro-radiological and cystoscopic techniques to manage complex ureteric stenoses or fistulae. We present a case in which this procedure was utilised, with an unusual intra-procedural finding. Macroscopic visualisation of lipids within the urinary tract is an unusual finding at CT, and rarely seen at cystoscopy. The aetiology of lipiduria is varied; it may indicate the presence of unrecognised pathology, occur as a sequelae of trauma, or arise secondary to intervention. Understanding the patient's history is vital to determining the likely aetiology and significance of such a finding.

## Clinical presentation

**Figure 1.**
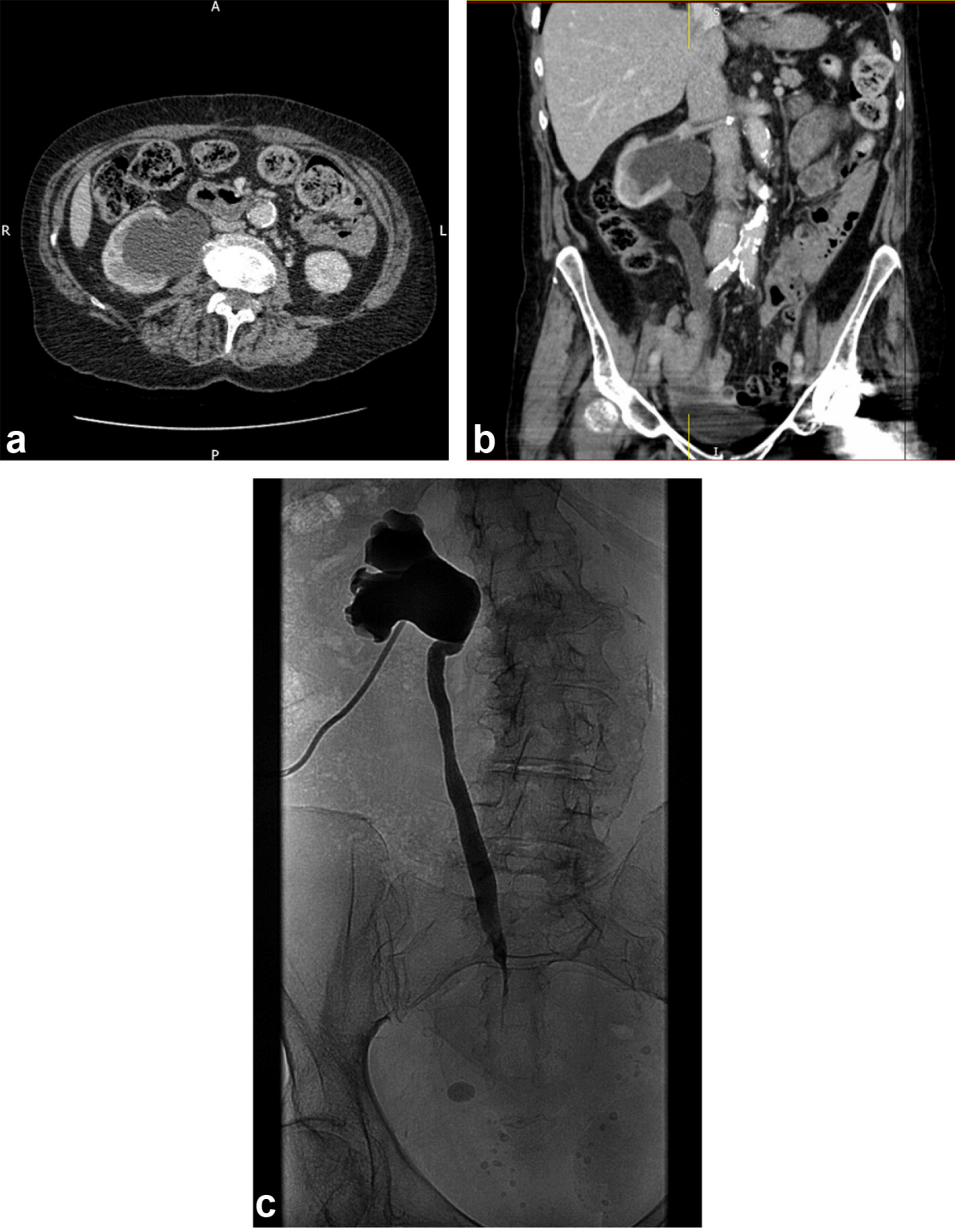
(a) Axial CT section at 6 months post operative, demonstrating marked right hydronephrosis and hydroureter (b). CT coronal reconstruction at 6 months post operative, demonstrating marked right hydronephrosis (1a) and hydroureter (1b). (c).Fluoroscopic renogram performed subsequent to CT (Figure 1) demonstrates right ureteric obstruction secondary to stricturing, likely to be at the site of reimplantation.

An 86-year-old female underwent elective surgery for a locally advanced rectosigmoid tumour with synchronous cervical tumour. A Hartmann's procedure was carried out, with total abdominal hysterectomy and bilateral oophorectomy. At the time of surgery the distal right ureter was found to be involved, and distal ureterectomy and reimplantation over a ureteric stent were also performed. The stent was removed at 3 months. Surveillance CT imaging at 6 months demonstrated marked hydronephrosis (Figure 1a) and hydroureter (1b). On fluoroscopic renogram (1c), this was proven to be proximal to a ureteric stricture at the site of reimplantation.

After failed attempts at antegrade stent insertion, temporising management was undertaken with nephrostomy, and the patient subsequently underwent cystoscopy as part of a combined endoscopic and uro-radiological rendezvous procedure to reinsert a ureteric stent.

**Figure 2.**
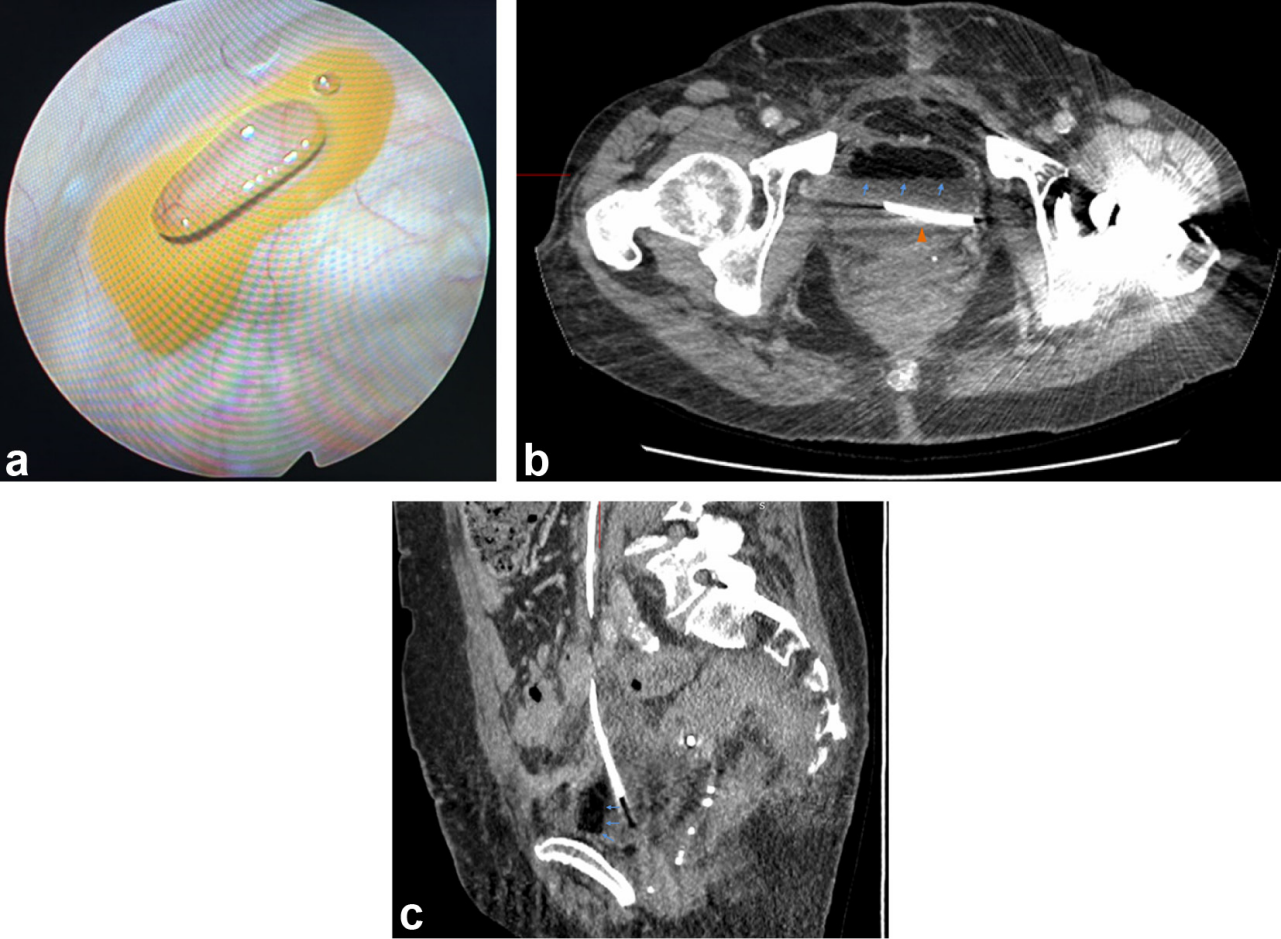
(a) Cystoscopic image demonstrating macroscopic fat in urinary bladder fundus. (b)Axial CT image at 6 months demonstrating intravesical fat-fluid level (arrows), correlating with the cystoscopic finding. Ureteric stent also noted (arrowhead). (c).Sagittally reconstructed CT image at 6 months demonstrating intravesical fat in the non-dependent position (arrows).

At cystoscopy, a surprising finding was made, with a large volume of viscous yellow fluid visualised lying anteriorly within the bladder (Figure 2a), which had the appearance of fat.

## Imaging findings

**Figure 3.**
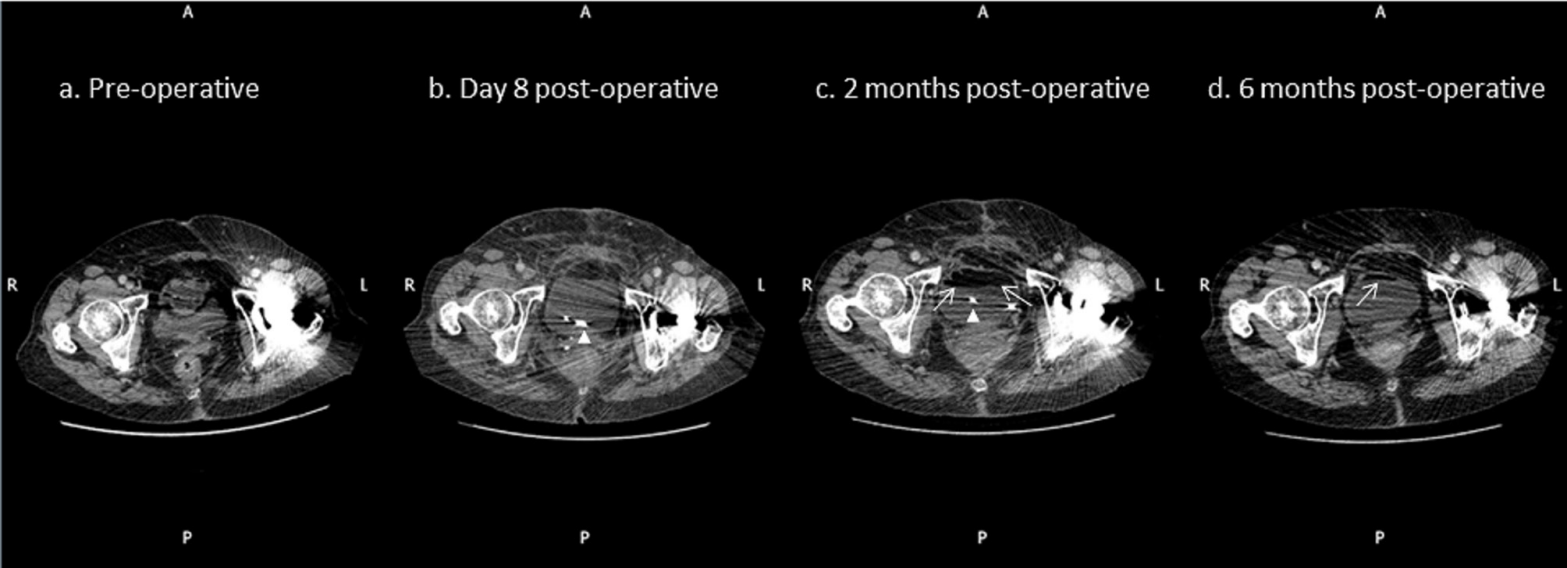
(a) Axial CT images through the pelvis at varying time points (pre-operative; 8 days, 2 months, and 6 months post-operative) demonstrating absence of fat/fluid level within the bladder (b).subsequent presence of intravesical fat as indicated by red arrowheads (c). and reduced volume at 6 months post-operative (d). (The patient’s ureteric stent has been removed).

Correlation with prior imaging revealed the presence of an intravesical fat-fluid level on CT carried out at 2 months post-operative, which had been overlooked in the report (Figure 2b,c). This was not present in either the patient’s pre-operative or early postoperative CT imaging performed at day 8 (Figure 3a,b). Subsequent CT imaging at 6 months also demonstrated the presence of an intravesical fat-fluid level, but with a smaller volume of the supernatant fat (Figure 3d).

## Treatment: The “Rendezvous” Procedure

Use of ureteric stents to maintain or restore ureteric patency is a well-documented and relatively common procedure for the management of both ureteric strictures and injuries, and indwelling ureteric stents are better tolerated than percutaneous nephrostomy.^1^ The procedure, whether antegrade or retrograde, requires the passage of a guidewire across the site of stricture and, as in our case, this may prove impossible. The ‘rendezvous’ procedure combines uro-radiological and endoscopic approaches, and often allows the negotiation of complex strictures or ureteric injuries with successful stent deployment, obviating the need for open surgical management or long-term percutaneous drainage.^1^ Whilst in the experience of our tertiary referral centre incidence of failure of either antegrade or retrograde stenting is rare, in such situations the rendezvous procedure provides a useful problem-solving management option.

**Figure 4.**
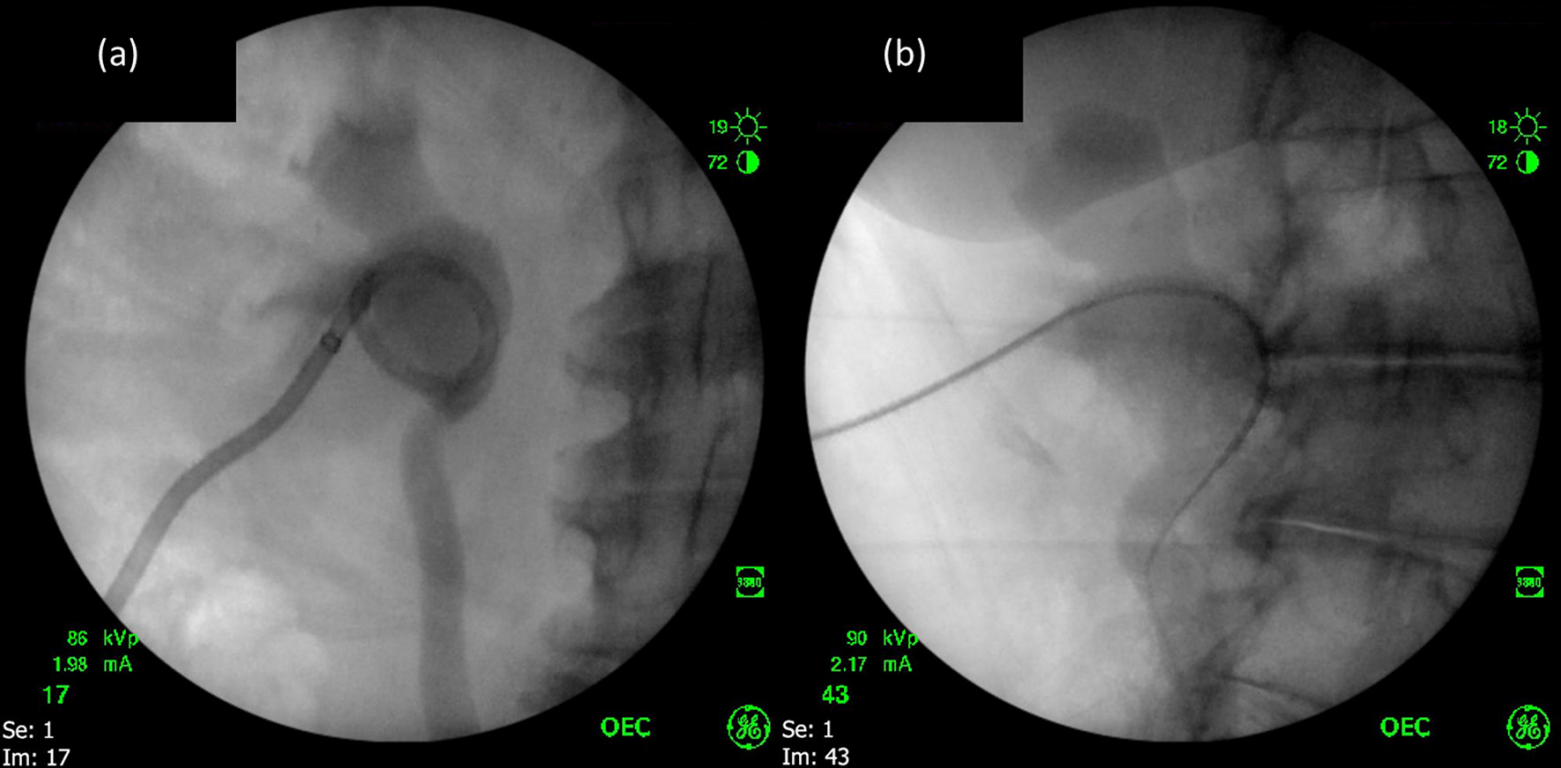
The patient’s nephrostomy (a) is exchanged over a guidewire for a selective catheter (b). Contrast medium is seen filling the right renal collecting system.

**Figure 5.**
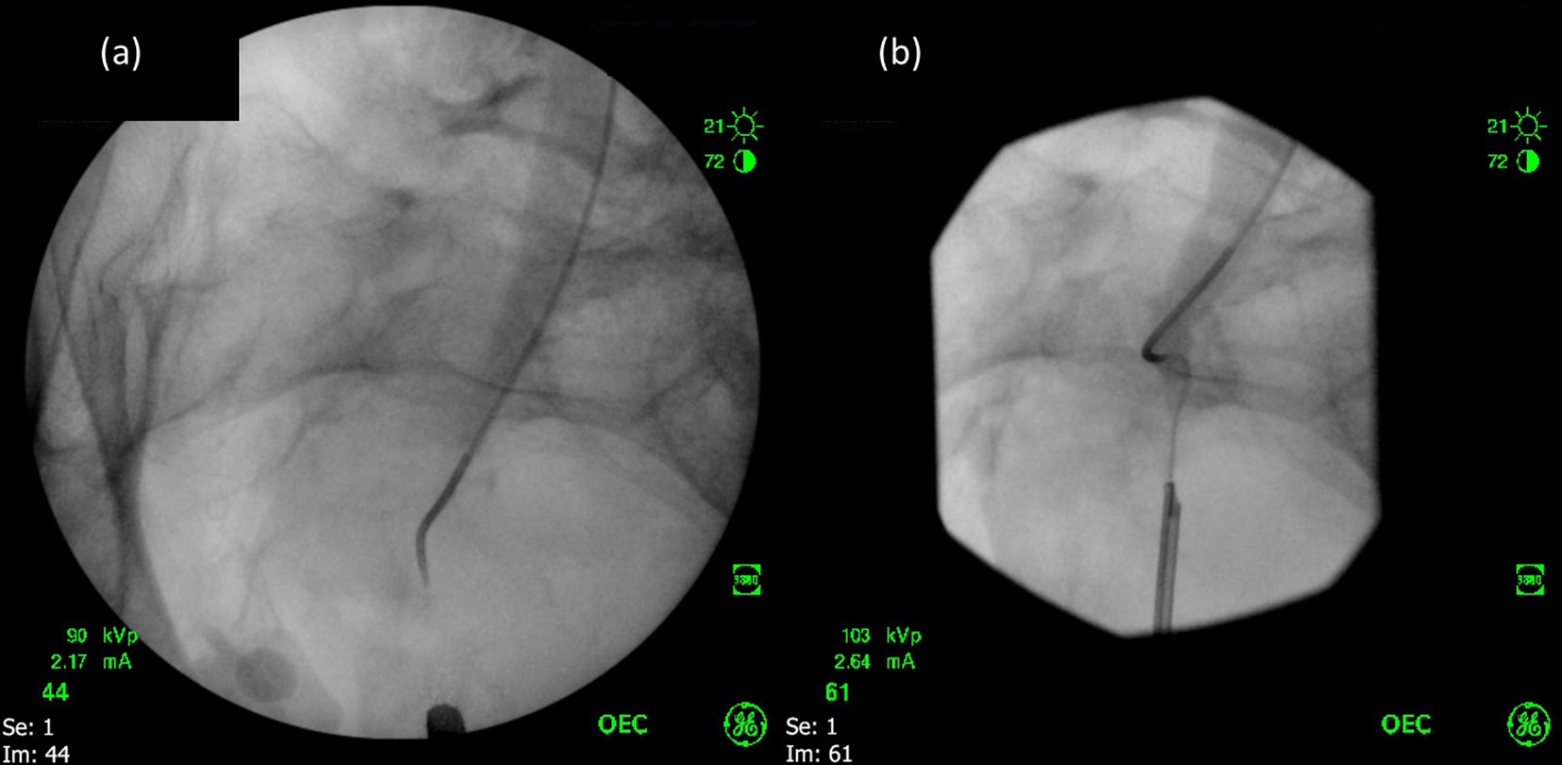
Under fluoroscopic guidance the guidewire and catheter are advanced to the proximal extent of the stricture whilst the ureteric orifice is accessed by ureteroscope (a). Using combined antegrade and retrograde approaches, the stricture is crossed (b).

**Figure 6.**
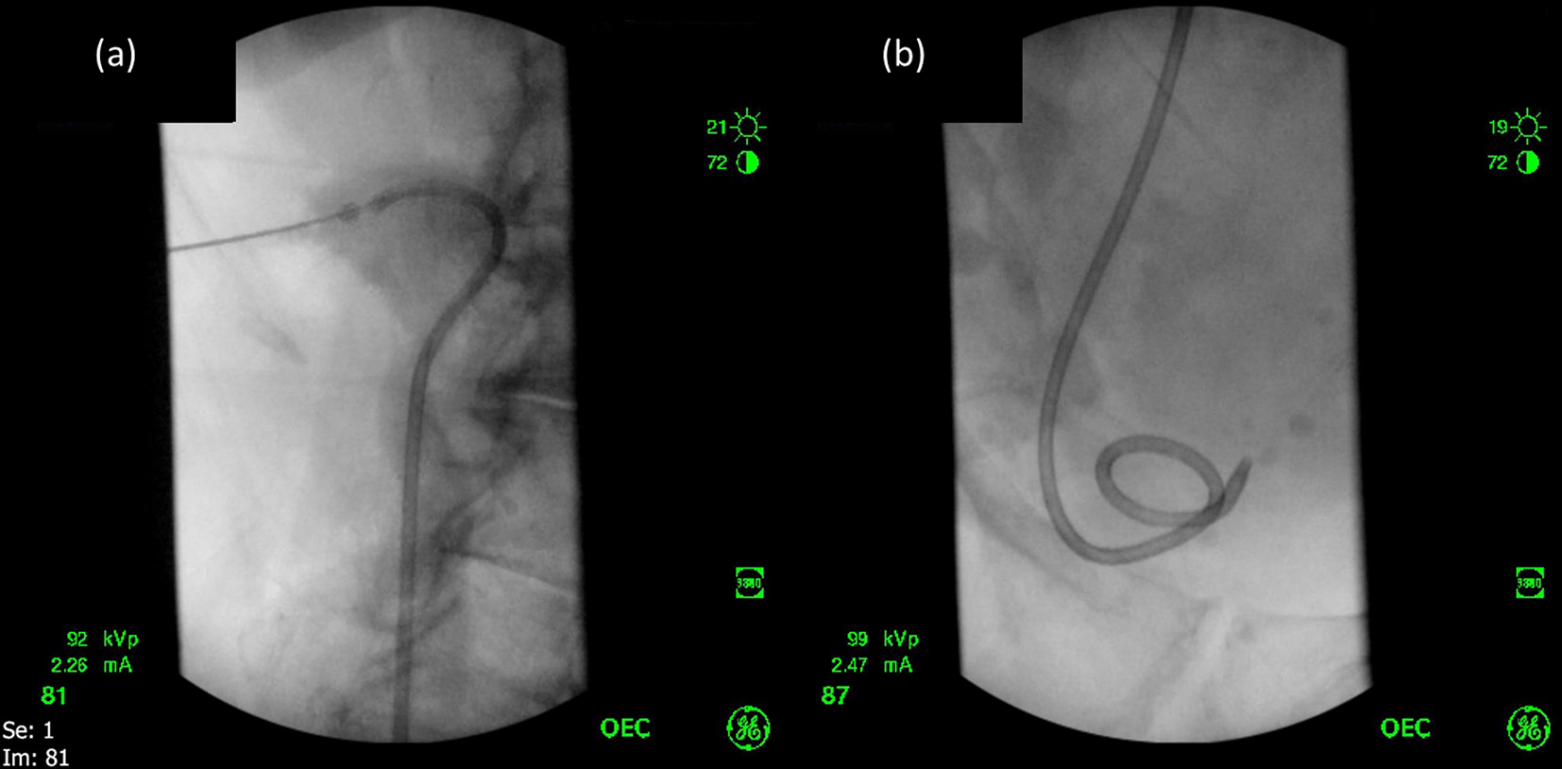
Subsequent placement of ureteric stent (6a), and (6b) Fluoroscopy demonstrates formation of the distal pigtail, confirming correct positioning of the distal tip of the stent within the urinary bladder.

The procedure is undertaken under general anaesthesia in an oblique supine position. The uro-radiologist exchanges the patient’s nephrostomy (Figure 4a) for a selective catheter (4b) which is advanced to the proximal extent of the stricture under fluoroscopic guidance, whilst concurrently the distal ureter is accessed by ureteroscope (Figure 5a). Contrast medium and methylene blue dye are mixed and instilled via the catheter, allowing a combination of fluoroscopic and direct visualisation of the stricture, which can subsequently be probed by guidewire from either antegrade or retrograde direction, or both (Figure 5b). Once the stricture has been crossed, dilators or a balloon catheter can be used to dilate to 9 French, allowing the placement of ureteric stent (Figure 6a,b).^1^

## Discussion

An intravesical fat-fluid level occurs with hydrophobic, low density fat molecules rising to the nondependent portions of the bladder above the denser normal bladder contents and seen on CT as a region of fat-density material (-60 to −120 Hounsfield Units) above the normal fluid-density (~1 Hounsfield Unit) content of the bladder, and best appreciated on sagittal reconstruction (as in Figure 2b).

Due to the contrast between fat and fluid, it is possible for the presence of fat in the vesicular cavity to be misinterpreted as gas. Although vesicular gas can be a sign of pathology, for example colovesical fistulation or trauma, it is more commonly seen following intervention such as urinary catheterisation. Use of lung or bone window pre-sets will allow fat and gas densities to be clearly distinguished, avoiding misinterpretation.

Intravesical fat-fluid level should also be distinguished from the “fat triangle sign” which has been described as imaging evidence of extraperitoneal bladder rupture, in which extravasated urine outlines an area of fat^2^; and from fat deposition in the bladder wall, which represents an uncommon but normal variant CT finding.^3^

Presence of hip prostheses, as in this case, can cause significant artefact on CT imaging due to photon starvation and beam hardening, with resultant reduced tissue contrast and spatial resolution, and associated increased risk of missed findings.^4^ Whilst beam hardening correction software can be used to minimise this artefact, in daily practice the use of increased window widths to review images (*e.g.* 3000–4000 HU window width, 800 window level) may allow improved visualisation of structures adjacent to metal hardware and reduce the effect of metal artefact.^5^

The presence of fat within the vesicular cavity can be divided into two entities, chyluria and lipiduria. The former represents the presence of microscopic lipid and cholesterol-rich lymph in the urine. This forms a milky-white emulsion and is due to abnormal flow of lymph from abdominal lymphatic system to the urinary tract,^5^ often secondary to a fistulous connection between lymphatic channels and urinary tract.^6^ Causes of chyluria are broadly classified as parasitic and non-parasitic, and it is a rare entity in developed countries, where it is generally of non-parasitic cause.^7^

Lipiduria refers to the presence of lipids in the urine, and can be a feature of nephrotic syndrome.^7^ It is a recognised sequelae of fat embolus,^8^ and has also been described following partial nephrectomy, renal transplant, and radiofrequency ablation of renal tumours.^9^ Whilst microscopic lipiduria may go unrecognised and therefore underreported, macroscopic lipiduria presenting as vesicular fat-fluid level at CT or, as in our patient, as visible fat within the bladder at cystoscopy, is more unusual. A number of cases have been reported, with differing aetiological hypotheses.

Martinez-Moya et al report a case of intravesical fat-fluid level secondary to traumatic bladder rupture, which was noted on the patient’s CT imaging at presentation.^10^ The proposed mechanism is of acute increase in intra-abdominal pressure leading to migration of small fatty droplets from the extravesical compartment through the ruptured bladder wall. Soussan et al have reported a case of fat-fluid levels demonstrated in the renal calyces following urine extravasation as a complication of high-grade urinary tract obstruction and infection, proposing a mechanism whereby extravasated urine induces lipolysis of perirenal fat, the components of which enter the renal collecting system and collect in the non-dependent position.^11^ Hoven et al presented a case in which a vesicular fat-fluid level appeared coincidentally with resolution of previously identified perivesical fat necrosis in a patient following right hemicolectomy. No bladder injury was reported, and transmural migration of lipids is suspected.^9^

Whilst it is possible that in our case a postsurgical lymphatico-ureteric or lymphatico-vesical fistula may have occurred, it is likely that this would result in chyluria, rather than frank lipiduria. It is also possible that urine extravasation occurred at the time of initial surgery, with consequential urine-induced lipolysis and subsequent migration of lipids either through the site of ureteric reimplantation, or through the bladder wall. We believe, however, that the most likely cause in our patient is that at the time of preparation of the ureter for reimplantation, a cuff of periureteric fat is likely to have been mobilised with the distal ureter and subsequently implanted within the bladder. Subsequent urine-induced lipolysis allowed the formation of the intravesical fat-fluid level, a feature rarely seen at cystoscopy.

## Learning points

- Presence of metallic hardware causes beam hardening artefact and resultant loss of tissue contrast at CT imaging, increasing the chance of missed pathology. Use of lung or bone window presets can help to minimise this risk, and is recommended as part of routine practice in the presence of metal prostheses or other such hardware.
- Fat within the urinary collecting system may be in the form of lipids, or in the form of a chylous emulsion, with distinct aetiologies for each.
- The presence of intravesical fat-fluid level on imaging may indicate the presence of unrecognised pathology, may be a sequelae of trauma, or secondary to intervention.
- Intravesical fat is an unusual finding at cystoscopy; lipiduria is a similarly uncommon CT finding, especially in the context of ureteric injury and reimplantation. Understanding the patient’s history is key to determining the likely aetiology of such findings.
- The combined urological ‘rendezvous’ procedure is a useful procedure employing both interventional radiology and cystoscopic techniques for the management of complex ureteric stricture or injury.
